# Pretreatment with Fucoidan from *Fucus vesiculosus* Protected against ConA-Induced Acute Liver Injury by Inhibiting Both Intrinsic and Extrinsic Apoptosis

**DOI:** 10.1371/journal.pone.0152570

**Published:** 2016-04-01

**Authors:** Jingjing Li, Kan Chen, Sainan Li, Tong Liu, Fan Wang, Yujing Xia, Jie Lu, Yingqun Zhou, Chuanyong Guo

**Affiliations:** Department of Gastroenterology, Shanghai Tenth People’s Hospital, Tongji University School of Medicine, Shanghai 200072, China; University of Manitoba, CANADA

## Abstract

This study aimed to explore the effects of fucoidan from *Fucus vesiculosus* on concanavalin A (ConA)-induced acute liver injury in mice. Pretreatment with fucoidan protected liver function indicated by ALT, AST and histopathological changes by suppressing inflammatory cytokines, such as tumor necrosis factor alpha (TNF-α) and interferon gamma (IFN-γ). In addition, intrinsic and extrinsic apoptosis mediated by Bax, Bid, Bcl-2, Bcl-xL and Caspase 3, 8, and 9 were inhibited by fucoidan and the action was associated with the TRADD/TRAF2 and JAK2/STAT1 signal pathways. Our results demonstrated that fucoidan from *Fucus vesiculosus* alleviated ConA-induced acute liver injury via the inhibition of intrinsic and extrinsic apoptosis mediated by the TRADD/TRAF2 and JAK2/STAT1 pathways which were activated by TNF-α and IFN-γ. These findings could provide a potential powerful therapy for T cell-related hepatitis.

## Introduction

Liver injury is the basis of acute liver failure. Severe and persistent liver damage eventually leads to liver failure. A number of factors, mainly viral infections, drugs, food additives, alcohol intake and radioactive damage can cause life-threatening disorders [1]. Among them, liver damage activated by T cells and macrophages is a common pathway [2].

Recently, the incidence of autoimmune and viral hepatitis which can develop into cirrhosis and even lead to death has risen sharply. The pathogenesis of these diseases is associated with the cytotoxic effect of sensitized T lymphocytes and pro-inflammatory cytokines produced by endotoxin-stimulated macrophages [2]. Currently, three types of inducers, including concanavalin A (ConA), D-galactosamine (D-GalN)/lipopolysaccharides (LPS) and high dose LPS were used to simulate the pathological processes in acute liver injury. Of these inducers, ConA which is characterized by easy establishment, obvious change in liver enzymes and cytokine release is the favored model to study the pathogenesis of liver injury [3]. ConA is a lectin purified from *Canavalia brasiliensis* [4]. In 1992, Tiegs and colleagues found that ConA had a special sugar-binding site essential for the induction of liver injury, however, subsequent research showed that ConA caused hepatocyte injury mainly through related cytokines produced by activation of CD4-positive T cells and macrophages [5]. The major cytokines involved in hepatitis development are tumor necrosis alpha (TNF-α), interferon gamma (IFN-γ), interleukin-2 (IL-2), and IL-6, of which TNF-α and IFN-γ are the dominant cytokines in irreversible biological damage [6–12]. These pro-inflammatory factors can lead to tissue bleeding and necrosis as a result of reduced lysosome stability or impaired endothelial cells as well as abundant nitric oxide via promotion of inducible nitric oxide synthase (iNOS).

Fucoidans, mainly from brown algae, are a class of polysaccharides containing sulfated fucose-rich residues [13]. In 1913, Kylin extracted “fucoidin” now known as fucoidan from marine brown algae, which was subsequently confirmed by Bird and Pervical [14–16]. Fucoidan from *Fucus vesiculosus* is an easily isolated basic component which was well characterized by Nishino [17]. Fucoidan is an effective compound which influences many pathological processes, and has attracted global interest. A large number of studies demonstrated that oral or intraperitoneal injection of 5–200 mg/kg fucoidan inhibited metastases and promoted apoptosis in colon, breast and lung cancers [18–20]. In hematopoietic progenitor cells, fucoidan showed an advantage in selectin inhibition. In particular, Granert and colleagues in 1999 demonstrated that fucoidan inhibited inflammatory infiltration [21], and in 2012 Kang and Cui showed the protective effect of fucoidan on lipopolysaccharide (LPS)-induced rat neuronal damage [22–23]. In addition, with regard to liver damage, ConA and D-galactosamine (GalN)-induced hepatopathy was improved, as indicated by Kawanno and Saito [24–25]. Common findings in these studies were the downregulation of related pro-inflammatory factors, including TNF-α, IFN-γ, IL-6, IL-8 and IL-12, and established the foundation of anti-inflammatory treatment. However, the significance of these studies was limited by surface phenomena and the mechanism of action of fucoidan was unclear. Establishing the clear mechanism of fucoidan provides an opportunity to study the pharmacological properties of potential effective drug candidates.

In this study, we investigated the mechanism of action of fucoidan in ConA-induced acute hepatitis. Based on the effect of fucoidan in reducing TNF-α and IFN-γ expression levels, we suspect that suppression of extrinsic and intrinsic apoptosis processes may be involved in the mechanism of action of fucoidan in liver protection.

## Materials and Methods

### Reagents

ConA and Fucoidan from *Fucus vesiculosus* were obtained from Sigma-Aldrich (St. Louis, MO, USA). Antibodies were provided by Cell Signaling Technology (Danvers, MA, USA) including the antibodies against iNOS, Bid, Bax, Bcl-2, Bcl-xL, TRADD, TRAF2, FADD, p-FADD, JAK2, p-JAK2, STAT1, and p-STAT1. The TNF-α and IFN-γ antibodies were purchased from Abcam (Cambridge, MA, USA) and Caspase 3, 8 and 9 were purchased from Proteintech (Chicago, IL, USA). The RNA polymerase chain reaction (PCR) kit was purchased from Takara Biotechnology (Dalian, China). Alanine aminotransferase (ALT) and aspartate aminotransferase (AST) microplate test kits were produced by Nanjing Jiancheng Bioengineering Institute (Jiancheng Biotech, China). The enzyme-linked immunosorbent assay (ELISA) kits were acquired from eBioscience (San Diego, CA, USA). The TdT-mediated dUTP nick end labeling (TUNEL) apoptosis assay kit was purchased from Roche (Roche Ltd, Basel, Switzerland).

### ConA-induced mouse model

In these experiments we used 84 male Balb/C mice (20–25 g, 6–8 weeks old) from Shanghai Laboratory Animal Co., Ltd. (SLAC, Shanghai, China). All animals were maintained under conditions of 22°C and 55% humidity with a 12 h light and 12 h dark cycle and monitored every day. All animal experiments were performed according to the National Institutes of Health Guidelines for the Care (Text A in S1 ARRIVE Checklist) and Use of Laboratory Animals and were approved by the Animal Care and Use Committee of Shanghai Tongji University, China. No animals died prior to the experimental endpoint or became severely ill. Throughout the course of this study, all efforts were made to minimize animal suffering.

Fucoidan was dissolved in saline at a daily dose of 10 mg/kg, 25 mg/kg and 50 mg/kg and administered orally for two weeks. ConA (20 mg/kg) was injected intravenously to induce hepatitis after fucoidan gavage. A total of 84 mice were randomly divided into six groups, as follows:

Group I, normal group (n = 6): saline

Group II, fucoidan group (n = 6): 50 mg/(kg·d) fucoidan

Group III, ConA group (n = 18): ConA injected via the tail vein

Group IV, ConA+fucoidan (n = 54): ConA+fucoidan (10 mg/kg, 25 mg/kg or 50 mg/kg)

Mice randomly selected from groups III and IV (6 mice at each dose) were anesthetized by an intraperitoneal standard dosage (1.25%) of sodium pentobarbital (Nembutal, St. Louis, MO, USA) at 2 h, 8 h or 24 h after ConA injection. The mice were decapitated after blood was drawn from the eyes and liver tissue were isolated and stored at -80°C.

### Determination of serum enzymes and cytokines

At different time points, blood was taken from the eyes and cryopreserved. A portion of serum was used for the determination of liver enzymes using the test kits and another portion was used to measure the levels of TNF-α and IFN-γ with commercial ELISA kits, according to the manufacturer’s instructions.

### Pathological assessments

Fresh liver tissue was fixed with 4% paraformaldehyde and dehydrated using different concentrations of ethanol. After immersing in xylene, the specimen was penetrated under constant temperature and packaged. The embedded tissue was cut at a thickness of 5 μm and stained with hematoxylin and eosin (H&E) for assessment by light microscopy.

### Detection of mRNA by quantitative real time PCR (qRT-PCR)

In order to analyze the gene expression of related factors, we extracted the total RNA and performed reverse transcription using TaKaRa kits, following the manufacturer’s instructions. SYBR Green quantitative real time PCR was performed to determine the expression intensity using the SYBR Premix EX Taq (TaKaRa Biotechnology, China) and detected by a 7900HT fast system (Applied Biosystems, CA, USA). The primer sequences are shown in Table 1.

**Table 1:** Nucleotide sequences of primers used for qRT-PCR.

Gene		Primer sequence (5'—3')
TNF-α	Forward	CAGGCGGTGCCTATGTCTC
	Reverse	CGATCACCCCGAAGTTCAGTAG
IFN-γ	Forward	GCCACGGCACAGTCATTGA
	Reverse	TGCTGATGGCCTGATTGTCTT
iNOS	Forward	GGCTTCACGGGTCAGAGCCA
	Reverse	TGCCCATTGCTGGGACAGTC
TRADD	Forward	GGCAGTGCATACCTGTTTTTG
	Reverse	AACCGCAACTGGACGATGAG
TRAF2	Forward	AGAGAGTAGTTCGGCCTTTC
	Reverse	GTGCATCCATCATTGGGACAG
Bcl-xL	Forward	ACATCCCAGCTTCACATAACCC
	Reverse	CCATCCCGAAAGAGTTCATTCAC
Bid	Forward	TCTGAGGTCAGCAACGGTTC
	Reverse	CTCTTGGCGAGTACAGCCAG
Bax	Forward	AGACAGGGGCCTTTTTGCTAC
	Reverse	AATTCGCCGGAGACACTCG
Bcl-2	Forward	GCTACCGTCGTCGTGACTTCGC
	Reverse	CCCCACCGAACTCAAAGAAGG
β-actin	Forward	GGCTGTATTCCCCTCCATCG
		CCAGTTGGTAACAATGCCATGT

### SDS-PAGE and Western blot analysis

The total protein extracted from cryopreserved tissue was separated by 8–12% sodium dodecyl sulfate-polyacrylamide gel electrophoresis (SDS-PAGE) and transferred onto polyvinylidene fluoride (PVDF) membranes. After blocking non-specific binding sites with 5% non-fat dried milk for 1 h, the membranes were incubated with primary antibodies overnight at 4°C. The antibody concentrations were as follows: Bcl-2 1: 500, Bcl-xL 1: 1000, Bax 1: 500, Bid 1: 500, Caspase-3 1: 500, Caspase-8 1: 1000, Caspase-9 1: 500, TNF-α 1: 500, IFN-γ 1: 1000, iNOS 1: 1000, JAK2 1: 1000, p-JAK2 1: 500, STAT1 1: 500, p-STAT 1: 1000, FADD 1: 500, p-FADD 1: 500, TRADD 1: 500, TRAF2 1: 1000 and β-actin 1: 1000. On the second day, the membranes were scanned using the Odyssey two-color infrared laser imaging system (fluorescence detection) after three washes and incubation (37°C for 1 h) with horseradish peroxidase-conjugated anti-rabbit or anti-mouse IgG (1:2000) to detect protein expression. The different molecular sizes were recorded by comparison with prestained molecular weight markers.

### Immunohistochemistry

Paraffin sections were completely melted in an electrothermal constant temperature oven and dewaxed in xylene. After dehydration with ethanol, endogenous peroxidase activity was blocked by 3% hydrogen peroxide solution. Citrate buffer (pH 6.0) was used to recover the antigens by cooling and heating repeatedly. The sections were then blocked with 5% bovine serum albumin (BSA) and incubated overnight with primary antibodies including TNF-α (1:100), IFN-γ (1:100), Bid (1:50), Caspase 8 (1:100), Caspase 9 (1: 50), TRADD (1:100), p-FADD (1:50), Bcl-2 (1:50), p-JAK2 (1:100), p-STAT1 (1:100). On the second day, the slices were washed three times and incubated with secondary antibodies. Diaminobenzidine (DAB) was applied to show positive particles. Color development was observed using a digital camera (Olympus) and calculated using Image-Pro Plus software 6.0 (Media Cybernetics, Silver Spring, MD, USA).

### Immunofluorescence

The dewaxed paraffin sections were blocked for 20 minutes by 5% normal donkey serum. Then, the sections were incubated overnight with primary antibodies including Bid (1:100), Caspase 8 (1:50), Caspase 9 (1: 50), Bcl-2 (1:100). PBS was used to wash the sections three times before and after incubation with the secondary antibody. Nuclear staining was performed by DAPI (1:1000). Fluorescence microscopy (Leica, Wetzlar Germany) was used to examine the green fluorescent cells.

### TUNEL staining

The prepared paraffin sections were dewaxed in xylene for 5–10 min twice and dehydrated with ethanol. Proteinase K without DNase was added at a concentration of 20 μg/ml for 15–30 min. TUNEL reaction buffer was dropped into the slices after being washed according to the manufacturer’s protocols. Apoptotic cells shown by a dark brown color were observed by optical microscopy and analyzed.

### Statistical analysis

Statistical analysis was performed with SPSS 20.0 software and evaluated by calculating the mean±SD. Quantitative data are representative of at least three experiments and were compared using the student’s t test and one-way analysis of variance (ANOVA). P values of <0.05 were considered statistically significant.

## Results

### Pretreatment with fucoidan ameliorated ConA-induced acute injury

**Fig 1:**
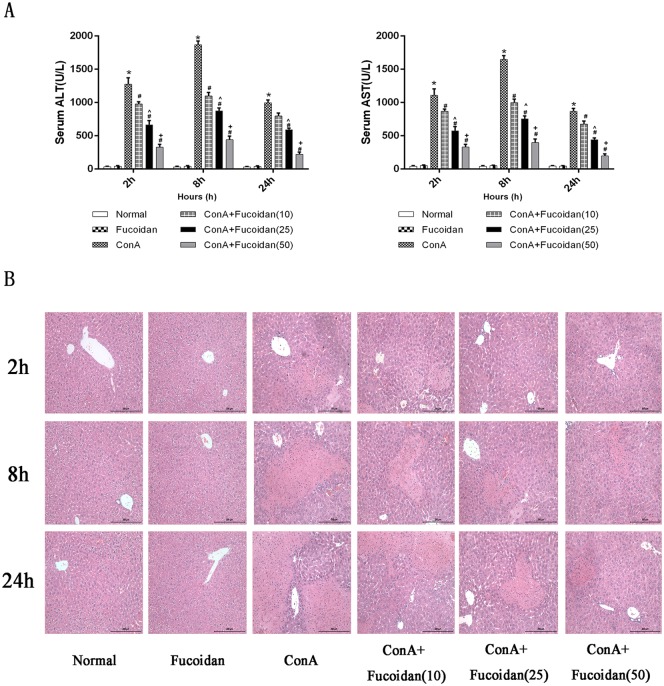
Effects of fucoidan on liver function and pathology in ConA-induced acute injury. (A) The mice were sacrificed at 2 h, 8 h and 24 h to collect blood and liver tissue. Serum ALT and AST levels were analyzed in the model group and fucoidan treatment groups (10 mg/kg, 25 mg/kg and 50 mg/kg). Data are shown as means ± SD. *P<0.05 for ConA vs Fucoidan, ^#^P<0.05 for ConA+Fucoidan (10, 25 and 50) vs ConA, ^P<0.05 for ConA+Fucoidan (25) vs ConA+Fucoidan (10), ^+^P<0.05 for ConA+Fucoidan (50) vs ConA+Fucoidan (25). (B) H&E staining was shown by digital microscopy. Original magnifications: 200×.

The results of serum ALT and AST, which were adopted to evaluate liver function in mice, indicated successful establishment of the injury model and showed a rapid increase (Fig 1A). However, the effects of fucoidan on liver enzymes were similar to those in the normal group. Satisfactory development of the ConA-induced injury model was observed in the treatment groups (10 mg/kg, 25 mg/kg and 50 mg/kg) in a dose-dependent manner at each time point. HE staining clearly showed edema and necrosis induced by the strong plant lectin. As shown in Fig 1B, a large area of edema appeared 2 h after ConA injection followed by necrosis. Decreased edema and necrosis were noted with increased fucoidan concentration at each time point, which demonstrated that fucoidan pretreatment effectively improved acute injury caused by ConA and protected liver cells from damage.

### Fucoidan pretreatment reduced the production of inflammatory factors in ConA-induced liver injury

**Fig 2:**
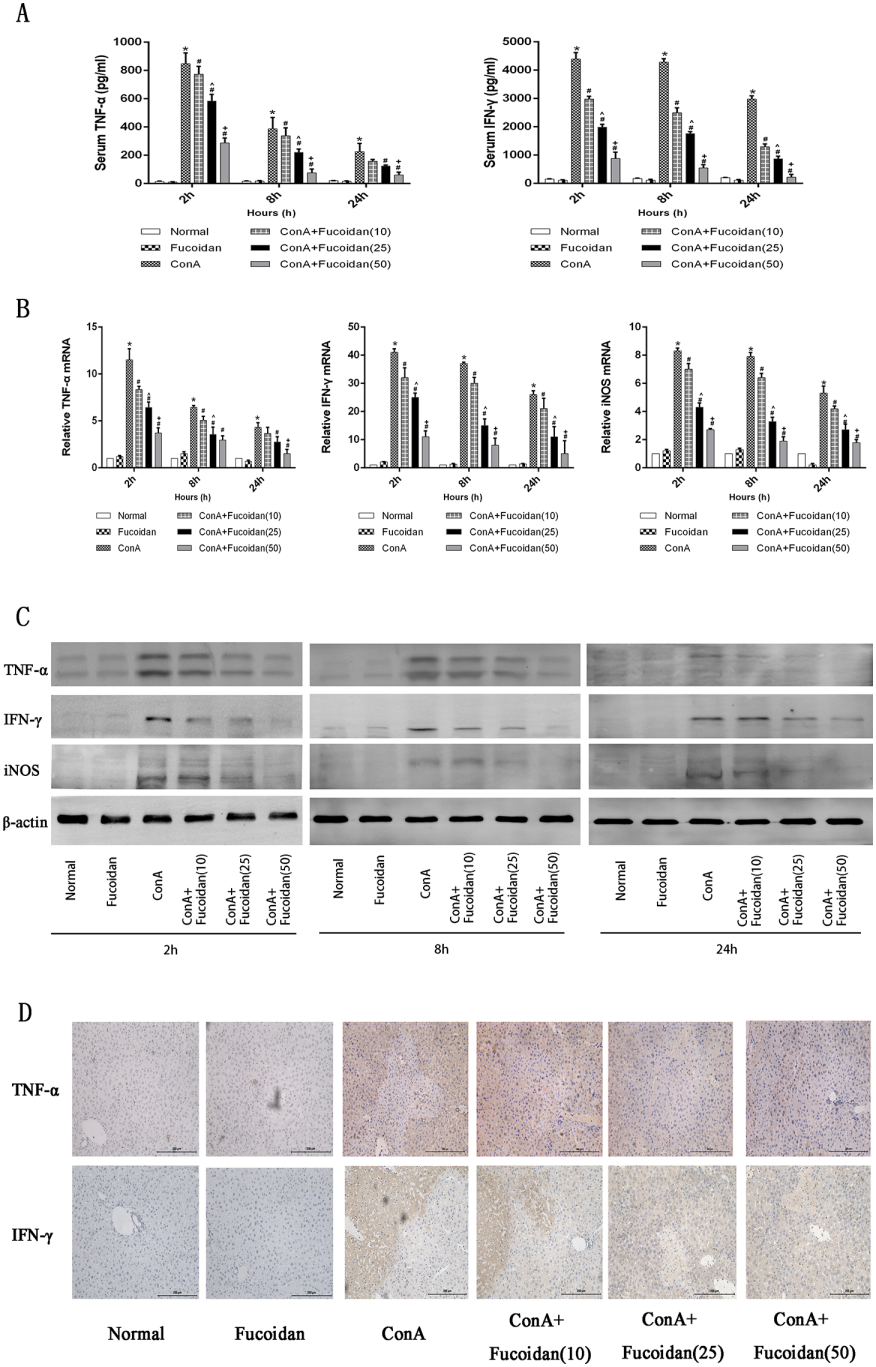
Effects of fucoidan on the production of inflammatory factors in ConA-induced acute liver injury. (A) The serum levels of inflammatory factors were measured by ELISA. (B) The expression of TNF-α, IFN-γ and iNOS mRNA levels were detected by real-time PCR. (C) The expression of TNF-α, IFN-γ and iNOS protein levels was measured by western blot. (D) Immunohistochemistry was carried out to evaluate TNF-α and IFN-γ. Original magnifications: 200×. The above data are shown as means ± SD. *P<0.05 for ConA vs Fucoidan, ^#^P<0.05 for ConA+Fucoidan (10, 25 and 50) vs ConA, ^P<0.05 for ConA+Fucoidan (25) vs ConA+Fucoidan (10), ^+^P<0.05 for ConA+Fucoidan (50) vs ConA+Fucoidan (25).

It is well known that the progression of hepatitis is closely associated with inflammatory factors including TNF-α, IFN-γ, IL-1β, IL-2, IL6 and iNOS. As the dominant factors in ConA-induced hepatitis, the levels of TNF-α and IFN-γ were detected by ELISA, real-time PCR, western blot and immunohistochemistry. Pure drugs had no effects on the release of pro-inflammatory factors, but ConA induced excessive production. TNF-α secretion reached a peak at 2 h and subsequently declined (Fig 2A and 2B). Other cytokines, such as IFN-γ and iNOS showed consistent results in PCR and western blot analysis. Different concentrations of drug treatment at all time points played a role in reducing inflammation damage by affecting both gene and protein expression (Fig 2C). The quantity of brown granules which represented target proteins also showed the excellent inhibitory effect of fucoidan (Fig 2D).

### Fucoidan pretreatment protects liver cells from damage by inhibiting intrinsic and extrinsic apoptosis

**Fig 3:**
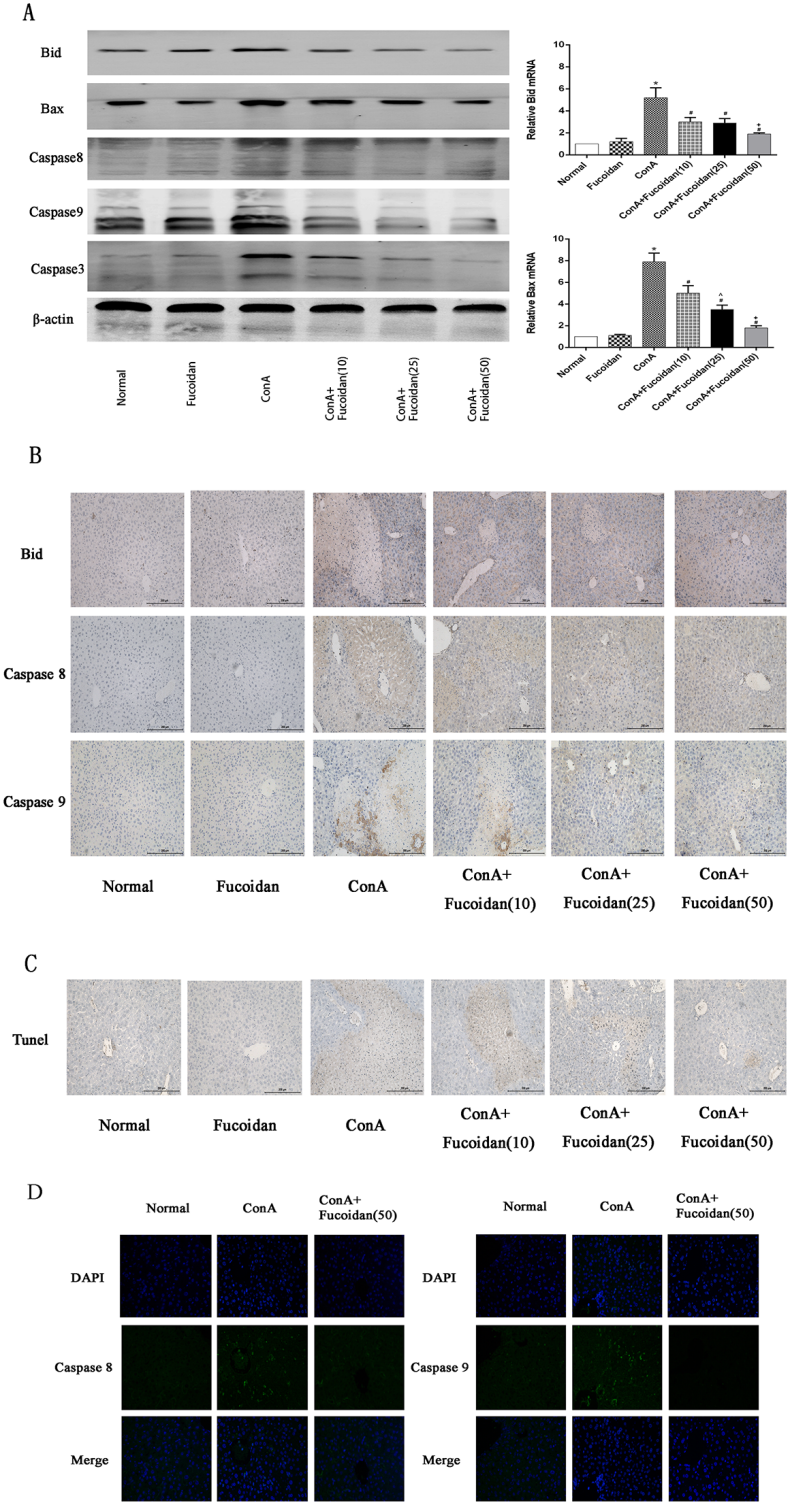
Effects of fucoidan on intrinsic and extrinsic apoptosis of ConA-induced acute liver injury. (A) The expression of Bid, Bax and Caspase 3, 8 and 9 at the gene and protein level were measured by PCR and western blot. Data are shown as means ± SD. *P<0.05 for ConA vs Fucoidan, ^#^P<0.05 for ConA+Fucoidan (10, 25 and 50) vs ConA, ^P<0.05 for ConA+Fucoidan (25) vs ConA+Fucoidan (10), ^+^P<0.05 for ConA+Fucoidan (50) vs ConA+Fucoidan (25). (B) Immunohistochemistry was used to evaluate Bid, Caspase 8 and Caspase 9. Original magnifications: 200×. (C) TUNEL staining of hepatic tissues at 8 h represents apoptotic cells. Original magnifications: 200×. (D) Immunofluoresence was used to evaluate Caspase 8 and Caspase 9. Original magnifications: 400×.

The effect of fucoidan on liver cell protection and the mechanism of action of the drug were explored further. We selected 8 h as the major time point in the following study. Marker proteins, such as Bid, Bax and caspase 9, of intrinsic apoptosis and caspase 8 which indicated extrinsic apoptosis were determined by western blot, PCR and immunohistochemistry. At 8 h, the pro-apoptotic proteins, Bid and Bax, were significantly increased at the gene and protein level which activated caspase cleavage to form cleaved caspase 9 and 8 that activated caspase 3-mediated cell apoptosis. Interestingly, fucoidan inhibited Bid, Bax and cleaved caspases at the doses used (Fig 3A). Immunohistochemistry and TUNEL staining also proved a dose-dependent decline in pro-apoptotic proteins and apoptosis with drug treatment (Fig 3B, 3C and 3D). The above results proved the effect of fucoidan on intrinsic and extrinsic apoptosis.

### Fucoidan pretreatment attenuated the TRADD/TRAF2 signal pathway by blocking the interaction between TNF-α and TRAF2

**Fig 4:**
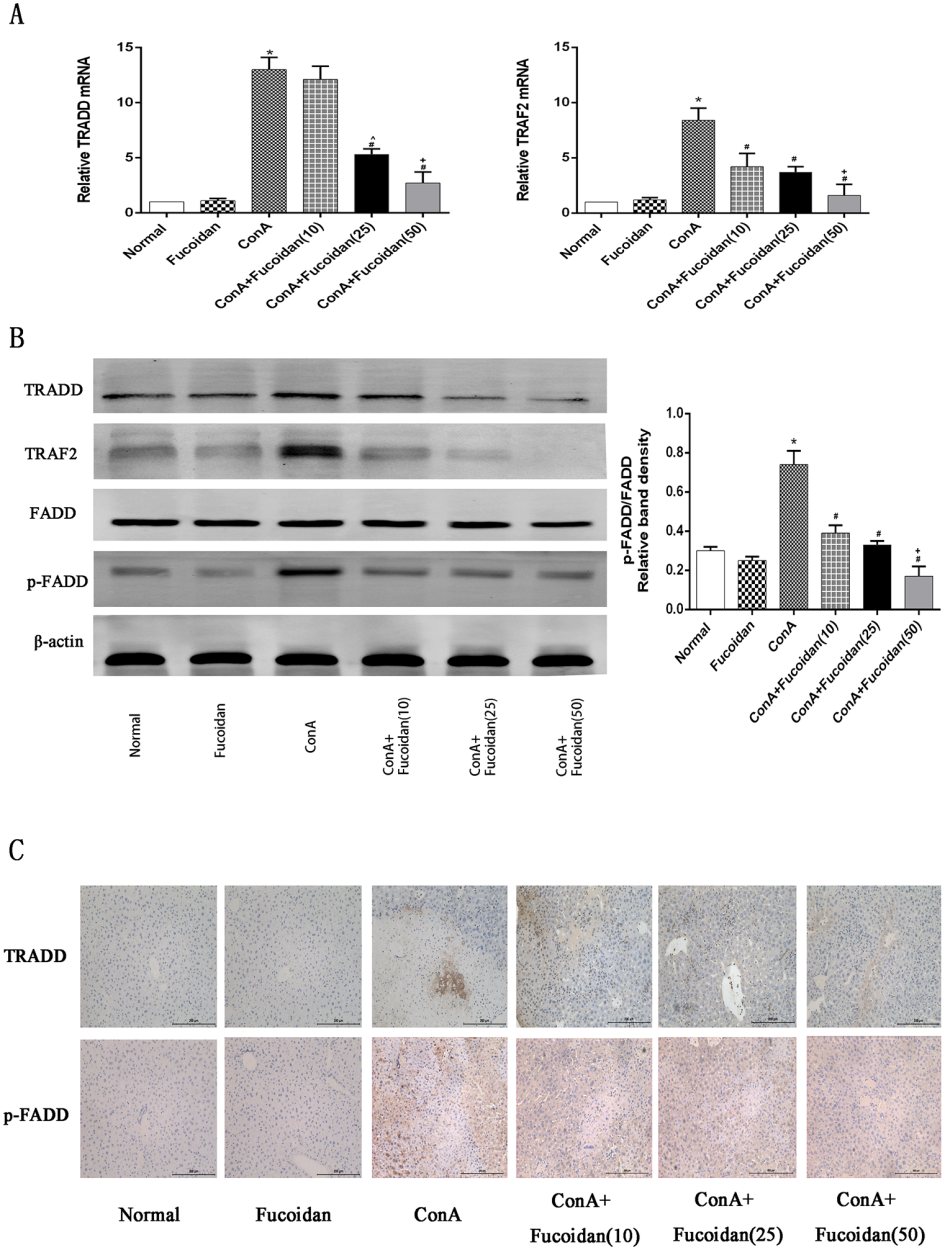
Effects of fucoidan on the activated TRADD signal pathway in ConA-induced acute liver injury. (A) The expression of TRADD and TRAF2 gene levels was measured by PCR. (B) The expression of TRADD, TRAF2, FADD and p-FADD protein levels was measured by western blot. The quantitative evaluation of p-FADD and FADD was determined by relative band density. (C) Immunohistochemistry was carried out to evaluate TRADD and p-FADD. Original magnifications: 200×. The above data are shown as means ± SD. *P<0.05 for ConA vs Fucoidan, ^#^P<0.05 for ConA+Fucoidan (10, 25 and 50) vs ConA, ^P<0.05 for ConA+Fucoidan (25) vs ConA+Fucoidan (10), ^+^P<0.05 for ConA+Fucoidan (50) vs ConA+Fucoidan (25).

Weakened apoptosis is a critical mechanism of fucoidan in the protection of ConA-induced liver injury. The dominant cytokine, TNF-α, has been shown to be the key in up-regulating apoptosis. To further clarify this process, we examined TRADD, TRAF2, FADD and p-FADD (Fig 4). TNF-α secretion activated by ConA increased the expression of TRADD and TRAF2 both at the gene and protein level and FADD phosphorylation was up-regulated. These phenomena demonstrated that ConA activated the TRADD/TRAF2 pathway which is associated with the two types of apoptosis. We then observed the change in mouse liver tissue after fucoidan pretreatment. A consistent decrease in the different drug treatment groups suggested that the TRADD signal pathway activated by TNF-α was attenuated by fucoidan via the inhibition of FADD phosphorylation.

### Fucoidan pretreatment attenuated JAK2/STAT1 pathway mediated Bcl-2 inhibition by blocking the release of IFN-γ

**Fig 5:**
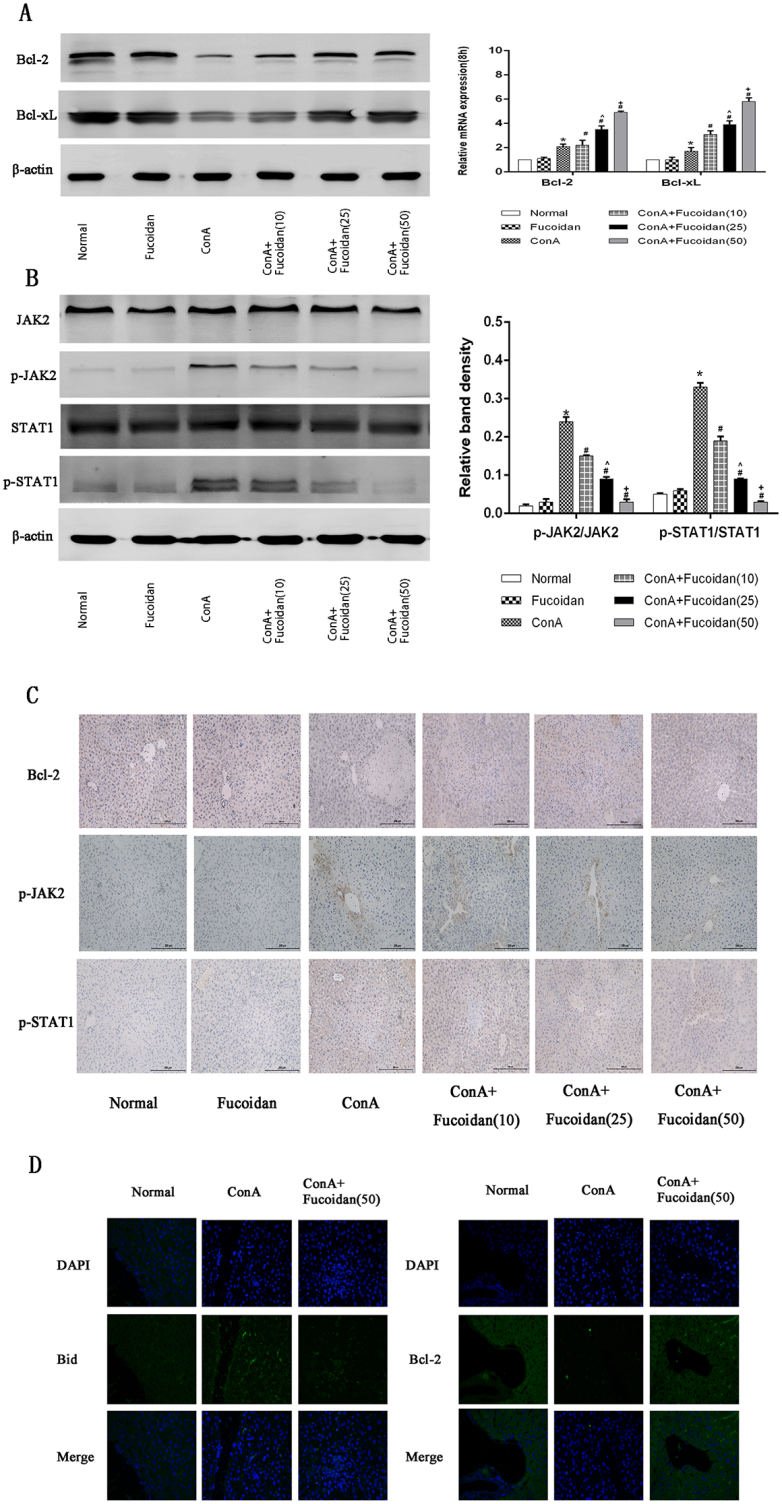
Effects of fucoidan on the activated JAK2/STAT1 signal pathway in ConA-induced acute liver injury. (A) The gene and protein expression of Bcl-2 and Bcl-xL were measured by PCR and western blot. (B) The protein expression of JAK2, p-JAK2, STAT1 and p-STAT1 was measured by western blot. The quantitative evaluation of p-JAK2/JAK2 and p-STAT1/STAT1 was determined by relative band density. (C) Immunohistochemistry was carried out to evaluate Bcl-2, p-JAK2 and p-STAT1. Original magnifications: 200×. (D) Immunofluoresence was used to evaluate Bax and Bcl-2. Original magnifications: 400×. The above data are shown as means ± SD. *P<0.05 for ConA vs Fucoidan, ^#^P<0.05 for ConA+Fucoidan (10, 25 and 50) vs ConA, ^P<0.05 for ConA+Fucoidan (25) vs ConA+Fucoidan (10), ^+^P<0.05 for ConA+Fucoidan (50) vs ConA+Fucoidan (25).

Another important inflammatory factor, IFN-γ, also plays an essential role in ConA-induced liver injury. It is commonly thought that the JAK2/STAT1 pathway can be activated by IFN-γ to inhibit the transcription of Bcl-2 and Bcl-xL. To assess the effects of fucoidan on the IFN-γ-related signal pathway, we assessed Bcl-2, Bcl-xL, JAK2 and STAT1 to prove a wider range of drug action. The anti-apoptotic proteins, Bcl-2 and Bcl-xL, showed reduced gene and protein expression after ConA injection and increased after fucoidan pretreatment (Fig 5A). The phosphorylation levels of key proteins including JAK2 and STAT1 showed a downward trend with increased fucoidan concentrations following abundant production activated by ConA (Fig 5B). Immunohistochemical staining showed that fucoidan not only had an effect on the TNF-α-related TRADD pathway, but also on the IFN-γ-mediated JAK2/STAT1 signal pathway (Fig 5C and 5D). Interaction between the two pathways suppressed apoptosis in ConA-induced acute liver injury.

## Discussion

Fucoidan, a stable bioactive ingredient found in a wide range of brown algae, has a diverse range of biological activities, which have been described in previous studies [13]. However, the underlying mechanisms are not well understood. The activation of T cells and macrophages stimulated by ConA is a superior model which imitates human viral and autoimmune liver diseases. We demonstrated that fucoidan pretreatment may protect against ConA-induced acute liver injury by inhibiting both intrinsic and extrinsic apoptosis using a well-established model and showed the significance of drug screening for T lymphocyte-dependent injury.

In our study, we verified the protective effects of fucoidan which is of great significance in our further research on ALT, AST and pathological changes. The inflammatory cytokines, TNF-α and IFN-γ, and iNOS produced by T cells and macrophages induced via ConA stimulation were promoted to release excess NO, which can cause large areas of pathological injury due to vasodilatation and other processes in organs, including the brain and spine [26–27]. In addition, TNF-α promotes the expression of other cytokines, such as IFN-γ and IL-6, combined with their corresponding receptors to cause leakage of enzymes resulting in a positive-feedback for iNOS secretion from macrophages in vitro and in vivo, as described by Do and Garcia [26, 28]. These effector molecules synergize to produce endothelial cytoskeleton remodeling or proteolytic enzyme release by mesenchymal cells resulting in cell edema and disorganization, dissolved and even necrotic hepatocytes. Degenerative changes in disruptive liver cells are involved in the sharp rise in serum ALT and AST associated with pathological damage [29]. As shown by our research results, ALT, AST and necrosis in the ConA group increased markedly at all-time points and declined after fucoidan pretreatment in a dose-dependent manner (10 mg/kg, 25 mg/kg and 50 mg/kg) based on no effect of fucoidan alone. These results conformed to the tendency of the related parameters TNF-α, IFN-γ, and iNOS detected by western blot, PCR and ELISA which demonstrated that fucoidan inhibited the production of inflammatory mediators to reduce liver damage. Other scientists have also described these findings in other systems, such as unclear gastric lesions, brain, and myocardium, and in acute hepatic injury [22–23, 30–31].

In addition to direct damage, apoptosis, a common phenomenon in acute liver injury, can affect individual cells in the population as opposed to large areas of necrosis. Various pathways activate apoptosis, such as Fas/FasL, NF-κB p65, ROS and MAPK families in ConA models [12, 32–36]. TNF-α, the main cytokine causing ConA injury, was considered to cause subsequent effects via its membrane receptor TNFR1 intracellular area, which contains a death domain structure and played an important part in cell dissolution [37]. In order to prove this conjecture, we selected the effective time point of 8 h to detect downstream effector molecules using a variety of molecular biological methods, such as PCR, western blot and immunohistochemical staining. The results showed that high expression of TRADD, TRAF2 and p-FADD in the ConA group was associated with elevated serum TNF-α and altered liver function. These parameters were reduced after drug treatment. These significant changes indicated that fucoidan firstly reduced serum TNF-α level and insufficient cytokines bound to TNFR1 to assemble TRADD, as demonstrated in a systematic analysis of the death domain of TRADD published in 1996 [38]. Next, the N terminal death effect structure of FADD raised by DD was unable to activate Caspase-8, a member of the cysteine-aspartic acid protease (caspase) family which executes cell apoptosis, as described by Jackson and Park [39–40]. On the one hand, caspase 8 can cause extrinsic apoptosis by activating caspase 3 which is responsible for PARP cleavage and DNA fracture. On the other hand, it also cracked Bid, a Bcl-2 family protein containing only the BH3 domain, to truncated (tBid) which enhanced membrane insertion of pro-apoptotic protein Bax after being transferred to the mitochondria [41–42]. Permeability of the mitochondria was changed by Bax and tBid to release cytochrome C that could activate caspase 9-induced intrinsic apoptosis [43–44]. According to previous research, determination of caspase 3, 8, 9, Bax, Bid and PARP related to the two types of apoptosis showed a satisfactory tendency in line with expectations. These results suggested that fucoidan may block the binding of TNF-α and its receptor TNFR1 to interfere with the activation of caspase 8, and then offers protection by inhibiting caspase 8 directly-induced extrinsic apoptosis and Bid/Bax-mediated intrinsic apoptosis.

**Fig 6:**
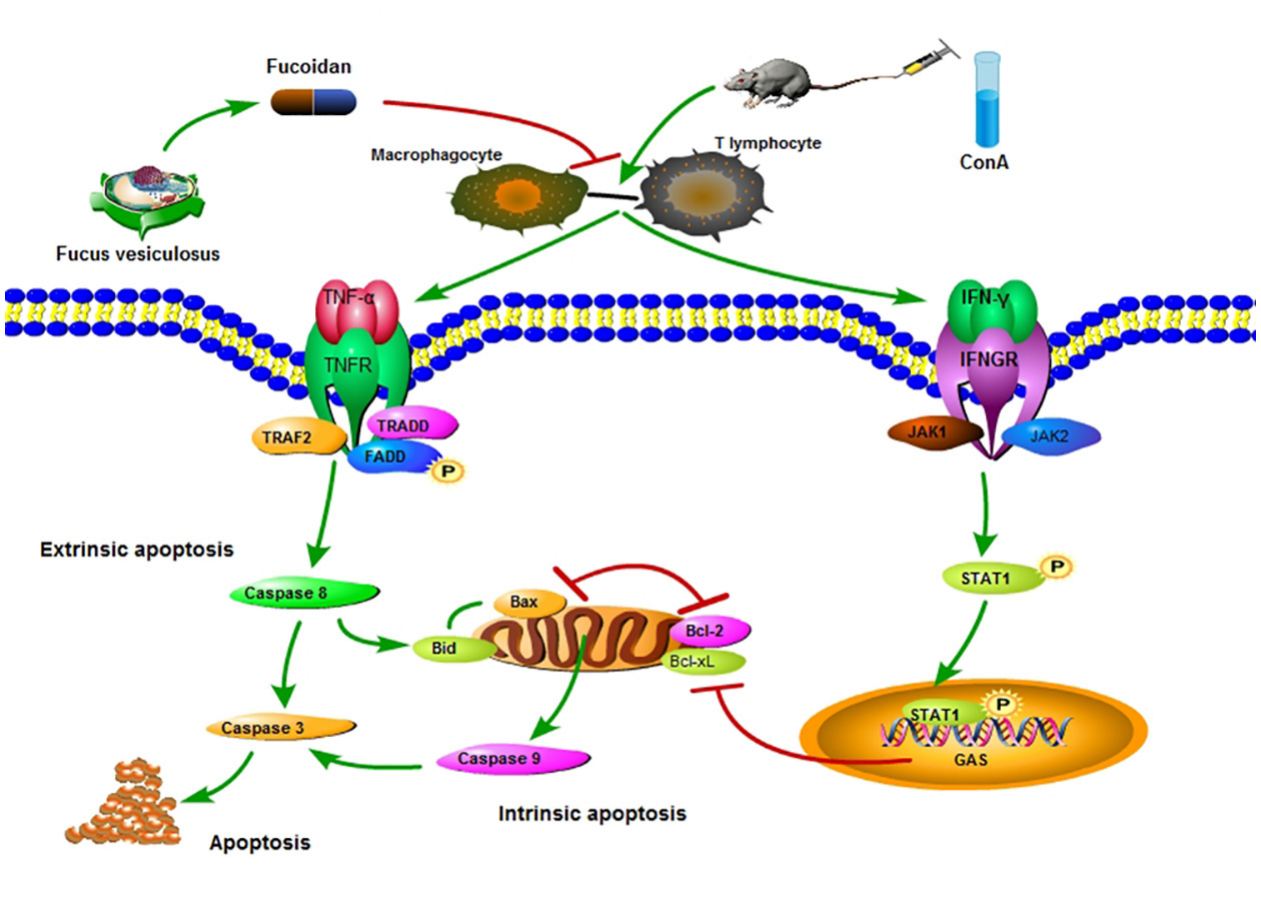
Mechanism of fucoidan action. In ConA-induced acute liver injury, overexpression of TNF-α and IFN-γ activate the TRADD/TRAF2 and JAK2/STAT1 pathways by combining with TNFR and IFNGR to regulate apoptosis. On the one hand, the phosphorylated FADD cleaved procaspase 8 which mediated extrinsic apoptosis. On the other hand, Bid transfer was induced by cleaved caspase 8 to activate caspase 9 with Bax which mediated intrinsic apoptosis. Another cytokine, IFN-γ, promoted apoptosis by inhibiting Bcl-2 and Bcl-xL expression mediated by the JAK2/STAT1 pathway. Thus, fucoidan successfully inhibited the release of TNF-α and IFN-γ in damaged liver cells to attenuate both intrinsic and extrinsic apoptosis by reducing the phosphorylation of FADD and STAT1.

Studies on T cell-dependent acute liver injury induced by LPS/GalN and ConA showed the strong cytotoxicity of IFN-γ which is an integral part in inducing tissue injury as well as hepatocyte apoptosis [8]. Firstly, IFN-γ is a inducer of iNOS which causes damage due to excess NO, and is an important activator of the JAK2/STAT1 pathway [45–46]. Gambin and colleagues reported that IFN-γ produced by macrophages combined with membrane receptors to activate JAK1 and JAK2 which recruited STAT1 [47–48]. Phosphorylated STAT1 was transferred from the cytoplasm into the nucleus to identify the gamma-interferon activation site (GAS) and promote intrinsic apoptosis by inhibiting transcription of anti-apoptotic proteins, Bcl-2 and Bcl-xL, located in the mitochondrial outer membrane [49]. High expression of STAT1 can also interact with TRADD to induce TNF-α-induced apoptosis [50–51]. IFN-γ in serum and tissue showed a significant reduction after fucoidan injection. The protective effect of fucoidan may be connected with the increase in anti-apoptotic proteins mediated by JAK/STAT1. This was proved by the analysis of Bcl-2, Bcl-xL, JAK2 and phosphorylated STAT1 levels in hepatic tissue. Therefore, we believe that fucoidan could play a protective role by reducing IFN-γ release which can promote apoptosis by inhibiting Bcl-2 and Bcl-xL expression mediated by the JAK2/STAT1 pathway (Fig 6). The mechanism of ConA-induced acute liver injury is difficult to completely explain due to its complexity and diversity and the evaluation of drug-related side-effects requires further study.

## Conclusions

In summary, our results showed that fucoidan attenuated acute liver injury induced by ConA via the suppression of both intrinsic and extrinsic apoptosis. The mechanism may be associated with the anti-inflammatory action of fucoidan identified by the reduction in TNF-α and IFN-γ release which inhibited the TRAF2/TRADD and JAK2/STAT1 pathways, respectively. The above results demonstrate that fucoidan may be a promising drug for T lymphocyte-dependent acute liver injury.

## Supporting Information

S1 ARRIVE Checklist NC3Rs ARRIVE Guidelines Checklist. The studies in vivo were in accordance with the ARRIVE guidelines, including how many mice were used, the sex of the mice, whether anesthesia and the method of sacrifice in the methods section of my manuscript.
(PDF)
